# Barriers and facilitators to sustainable operating theatres: a systematic review using the Theoretical Domains Framework

**DOI:** 10.1097/JS9.0000000000000829

**Published:** 2023-10-26

**Authors:** Aws Almukhtar, Carys Batcup, Miranda Bowman, Jasmine Winter-Beatty, Daniel Leff, Pelin Demirel, Talya Porat, Gaby Judah

**Affiliations:** a Department of General Surgery, Imperial College Healthcare NHS Trust; b Department of Surgery and Cancer, St Mary’s Hospital; c Dyson School of Design Engineering, Imperial College London; d Department of Breast Surgery, Imperial College Healthcare NHS Trust, Charing Cross Hospital, London, UK

**Keywords:** behavioural determinants, carbon emissions, environmental sustainability, healthcare, operating theatres, surgery, Theoretical Domains Framework

## Abstract

**Background:**

The health sector contributes significantly to the climate crisis. Operating theatres (OTs) in particular are a major contributor of greenhouse gas emissions and waste, and while there are several evidence-based guidelines to reduce this impact, these are often not followed. The authors systematically reviewed the literature to identify barriers and facilitators of sustainable behaviour in OTs, categorising these using the Theoretical Domains Framework (TDF).

**Materials and methods:**

Medline, Embase, PsychInfo, and Global Health databases were searched for articles published between January 2000 and June 2023, using the concepts: barriers and facilitators, sustainability, and surgery. Two reviewers screened abstracts from identified studies, evaluated quality, and extracted data. Identified determinants were mapped to TDF domains and further themes as required. The results were reported in line with PRISMA (Preferred Reporting Items for Systematic reviews and Meta-Analyses) and AMSTAR (A MeaSurement Tool to Assess Systematic Reviews) guidelines.

**Results:**

Twenty-one studies were selected for analysis and assessment (17 surveys and four interview studies) comprising 8286 participants, including surgeons, nurses, and anaesthetists. Eighteen themes across 10 TDF domains were identified. The most common barriers to adoption of green behaviours in OTs were in domains of: ‘knowledge’ (*N*=18), for example knowledge of sustainable practices; ‘environmental context and resources’ (*N*=16) for example personnel shortage and workload and inadequate recycling facilities; ‘social influences’ (*N*=9) for example lack of leadership/organisational mandate or support; ‘beliefs about consequences’ (*N*=9) for example concerns regarding safety. Intention was the most common facilitator, with 11 studies citing it.

**Conclusions:**

Despite intentions to adopt sustainable practices in OTs, this review identified several barriers to doing so. Interventions should focus on mitigating these, especially by improving staff’s knowledge of sustainability practices and working within the environmental context and time pressures. Furthermore, institutional change programmes and policies are needed to prioritise sustainability at the hospital and trust level. Additional qualitative work should also be conducted using behavioural frameworks, to more comprehensively investigate barriers and determinants to decarbonise OTs.

## Introduction

Highlights

This review found that the main barriers cited in the literature to green surgery are:

- Lack of knowledge (of sustainable practices or sustainability goals).
- Context and resources (e.g. staffing, time pressures, or inadequate recycling facilities).
- Lack of organisational support was also cited as a barrier, yet there was evidence of intentions to make proenvironmental changes.

Recommendations:

- Interventions should be co-designed with theatre staff to address these determinants in a feasible and effective way, e.g. by training theatre staff on sustainable behaviour and creating a ‘green culture’.
- Additional in-depth work, based on behavioural science frameworks, to give a deep and nuanced understanding of the barriers and facilitators from various stakeholders in the operating theatre context. More detailed and specific knowledge would better inform interventions facilitating sustainable behaviours with minimal effort (e.g. replacing single-use equipment with reusable) or by facilitating informed decisions (e.g. labelling packages with explicit waste instructions, for example: recycle).

Solutions informed by a full understanding of the most important contextual barriers and facilitators are more likely to be effective, and will also address the inevitable barriers of time, personnel shortages, and the need for convenience.

Improving sustainable and proenvironmental practices is key to combating climate change. Healthcare has a substantial influence on carbon emissions and other environmental issues, for example through waste generation (e.g. disposable items)^1,2^, use of anaesthetic gases^3–7^, the vehicles involved in patient transport^2,8^, water^2,9^, and electricity use^8,10,11^. A report by Healthcare Without Harm^12,13^ estimates that the carbon footprint of healthcare equates to 4.4% of global net emissions. In recent years there have been initiatives by governments and institutions across the world to attempt to reduce this impact, for example the NHS has set the goal of net-zero emissions by 2045^14^. In order to achieve this goal, the NHS has included sustainability in its healthcare plans for a decade^15,16^. However, the challenge is enormous—in order to reach its target, the NHS will need to remove the equivalent of the emissions of Croatia from its current carbon footprint^15^.

The environmental impact of healthcare is distributed unevenly across the system. Studies using life cycle assessments have determined the ‘carbon hotspots’, which represent areas of high environmental impact and are exceedingly important targets for improvement^7,17–19^. These services include surgeries, MRI services^11^, and dialysis^8,20^. Within the surgical services, the operating theatre has been consistently quoted as a highly resource-intensive and therefore a high environmental impact area of healthcare^3,10,21–23^. Studies looking at the impact of various operations, such as cataracts, hysterectomies, and dermatologic procedures, have identified common sources of high emissions; particularly, single-use surgical tools, energy needed for ventilation and heating/cooling, and anaesthetic gas use^3,21,22^. Moreover, studies show that operating theatres (OTs) are 3–6 times more energy-consuming than the rest of the hospital and are responsible for 21–30% of hospital waste^3,24–26^. Since OTs often have their own supply chains, represent a physical area of the hospital under separate managerial control, and are operated by defined professional groups, strategies targeting environmentally sustainable behaviours could be most impactful within healthcare. OTs also represent one of the most stringent areas for infection control protocols which can challenge some sustainability initiatives (e.g. reusables). Reviewing the case of OTs provide opportunities to roll out behavioural change with ease in other parts of the hospital with less stringent infection control requirements.

Previous systematic reviews have investigated how the impact of the operating theatres on the environment could be reduced^23,27–30^. These reviews concluded that many strategies to reduce environmental impacts rely on ongoing behavioural changes^6,23^. Therefore, many interventions to reduce the environmental impact of operating theatres relate to behaviour change, such as the use of regional or local anaesthesia rather than general^27^, scrubbing with alcohol instead of handwashing^23^, and dispensing of waste appropriately^22^. Additionally, this growing body of evidence, coupled with healthcare emission targets, and enthusiasm from clinicians^23,31,32^ have led to the creation of guidelines and suggestions for reducing the environmental impact of operations^33,34^. For instance, the Royal Colleges of Surgeons of Edinburgh, England, and Glasgow have created the ‘Intercollegiate Green Theatre Checklist’, which identifies specific, evidence-based and practical areas for improvement for surgeons^35^. The checklist encourages certain behaviours, such as opening only the instrument sets that are needed, powering off ventilation after surgery, and checking anaesthetic gas equipment for any leaks^35^. Moreover, organisations such as Practice Greenhealth^36^ and the international HealthCare Without Harm^37^, which are dedicated to reducing the impact of healthcare on the environment, advocate for practices such as minimising waste, conserving water and energy, and using less impactful anaesthetic gases for operations^38^.

Despite the growing number of guidelines and recommendations aimed at reducing the impact of OTs, these are rarely followed^39^. Some of the reasons for this are lack of structural support^8^, the context of the workplace (e.g. incorrect recycling bins)^40,41^, having an operating theatre ‘routine’ already in place^38^, lack of time^40^, and focus on patients’ priorities over environmental issues^38,42^. It is necessary to understand the barriers and facilitators to the adoption and implementation of sustainability guidelines and recommendations, in order to design interventions which can effectively increase sustainable practices^43^.

The use of a behavioural framework can facilitate a comprehensive assessment of behavioural determinants according to recognised categories. The Theoretical Domains Framework (TDF) consists of 14 domains which were determined through consolidating domains from 33 behaviour change theories in a process of expert consensus^44^. The TDF has been used extensively in studies of behaviour change as it provides a comprehensive framework for understanding the factors influencing behaviour. This is especially true for systematic reviews of behaviour change in healthcare, where it is a useful tool for coding behavioural determinants and comparing studies in a meaningful way^45–47^. The TDF also fits within a wider process for understanding and changing behaviour, including the Behaviour Change Wheel^48–50^ and the Behaviour Change Techniques Taxonomy^51^. Therefore, it can be used in combination with these other models and tools in order to create appropriate interventions which address the relevant determinants^52–54^.

This review aimed to identify the barriers and facilitators to behaviours which reduce the environmental impact of OTs. We used the TDF to analyse the data collected through the systematic search of the literature^54^. This categorisation using the TDF can inform the design of future behavioural interventions, which would aid to reduce carbon emission of OTs, and by extension, healthcare.

## Methods

The study protocol was established prior to starting the conduct of the review and was registered retrospectively^55^. A systematic electronic search was carried out following the principles outlined by the Cochrane Collaboration^56^ in Medline, Embase, PsychInfo, and Global Health from January 2000 until June 2023 using Ovid. Screening, data extraction, and reporting followed the Preferred Reporting Items for Systematic reviews and Meta-Analyses (PRISMA) guidelines and recommendations^57^ and obtained a moderate quality when assessed using the AMSTAR (A MeaSurement Tool to Assess systematic Reviews) guidelines^58^.

### Eligibility criteria

Table 1 summarises the inclusion and exclusion criteria. Studies were included if they discussed barriers and/or facilitators to the adoption of more environmentally sustainable behaviours in OTs, were published during or after the year 2000, in English, and as a peer-reviewed journal article. There was no restriction on study methodology or randomisation.

**Table 1:** Inclusion and exclusion criteria for the search.

Inclusion	Exclusion
Population: healthcare staff, aged over 18	Population: nonsurgical staff or patients
Barriers or facilitators to adoption of green surgery guidelines	–
Setting: operating theatres	Setting: nonsurgical specialty or outside operating theatres, e.g. surgical wards
Studies published during or after 2000	Studies published before 2000
Study designs: any	Commentaries, Editorials, Letters, Abstract only published, Conference posters, and Case report
Study language: English only	Language other than English

### Search strategy

The search strategy was formulated with the help of a medical librarian. It contained both Medical Subject Heading (MeSH) and non-MeSH terms combined using Boolean logic strings. The following concepts were combined: barriers and facilitators, sustainability, and surgery. See Table 2 for the search terms used.

**Table 2:** Summary of search terms used.

	Non-MeSH terms		
MeSH terms	Barriers and facilitators	Sustainability	Surgery
Attitude physician attitude attitude to change carbon footprint greenhouse gas recycling environmental protection surgery	Barrier? facilitat* motivat* attitude? enable* knowledge determinant* hurdle? Obstacle?	Environment* adj2 sustainab* life cycle carbon adj2 footprint* ecolog* adj2 footprint environment* adj2 pollution* waste adj2 management waste adj2 reduction waste adj2 stream* recycl* reuse reusable waste adj2 reduc*	Surge* Surgical Operation* adj2 theatre* Operat* adj2 Theatre* Operat* adj2 room

MeSH, Medical Subject Heading;

Truncation symbols (*), used as a substitute for any string of zero or more characters at the end of a word;

Wildcard symbol (?) can be used as a substitute for one character or none;

Adj2, adjacency searching for both terms and up to one word in between them.

We used ‘AND’ conjunction between the different three categories using MeSH and Non-MeSH terms: ‘Barriers and facilitators’ terms AND ‘Sustainability’ terms AND ‘Surgery’ terms.

### Study selection

The studies were uploaded onto Covidence systematic review software^59^, an online tool which supports the study screening process. Two authors out of a team of three independently screened the title and abstract of each study to determine its suitability for inclusion. Two authors are clinicians working in OTs with research experience; one author is a behavioural researcher. If there were conflicts in the decision made, these were resolved through discussion or the involvement of a senior author, a behavioural researcher. The full texts of selected papers were screened by two authors, and final decisions were made on inclusion through discussion, based on the eligibility criteria.

### Data extraction

The reviewers extracted the data from the included papers using a pre-agreed data extraction template in Excel. As highlighted in Table 3, data extracted included: year of publication, title, country, study type (e.g. qualitative), aim, sample size, description of the population (e.g. perioperative nurses), sample age average and variance, data collection method, analysis used, and environmental behaviour investigated (e.g. recycling).

**Table 3:** Study characteristics.

References	Design	Country/ies	Participants	Study aims	Focus area
Ard *et al*. (2016)^60^	Survey (quantitative)	US	2036 anaesthetists	Understand the current state of environmental practice, attitudes, and knowledge among anesthesiologists in the United States	General sustainability
Azouz *et al*. (2019)^61^	Survey (quantitative)	US	524 theatre staff	Identify barriers to OR recycling and implement a recycling improvement educational programme	Recycling
Burbridge *et al*. (2019)^62^	Interviews (qualitative)	US	Draëger (anaesthesia workstation manufacturer) product representatives, anaesthesiologists, anaesthesia technicians, perioperative nurses, hospital waste management personnel, product manufacturers. Numbers not reported	Identify opportunities and barriers for recycling and waste reduction in disposal of exhausted CO2 absorbers	Recycling Waste reduction
Chang and Thiel (2020)^63^	Survey (quantitative)	US	1634 surgeons and nurses, including 1262 cataract surgeons and 301 nurses and administrators for cataract surgery	Evaluate opinions regarding OR waste, factors that drive excessive waste, and willingness to consider economic and environmental sustainability initiatives	Waste reduction
Chang *et al*. (2023)^64^	Survey (quantitative)	US	458 opthalmic surgeons	Evaluate opinions regarding OR waste, factors that drive excessive waste, and opinions Regarding Reuse of Surgical Products, Pharmaceuticals, and Instruments	General sustainability
Conrardy *et al*. (2010)^65^	Survey (qualitative and quantitative)	US	172 surgeons and surgical technologists	Evaluate whether reusable supplies would meet the same high standards as disposable supplies and reduce the regulated waste stream in two ORs	Reuasables vs single-use items Waste reduction
Fraifeld *et al*. (2021)^66^	Survey (quantitative)	US	70 anaesthetists: 10 anaesthesiologists, 51 nurses, 9 anaesthesia technicians	Assess anaesthesia staff knowledge, attitudes, and practices	General sustainability
Frewen *et al*. (2022)^67^	Survey (quantitative)	South Africa	222 anaesthetists	Assesses the opinions and knowledge of South African anaesthetists regarding the environmental impact of anaesthetic practice	General sustainability
Harris *et al*. (2021)^68^	Survey (qualitative and quantitative)	UK and Ireland	150 surgeons: 72 consultants, 35 specialist trainees, 22 core trainees and 1 clinical fellow	Explore the current attitudes and behaviours of surgeons and surgical trainees towards environmental sustainability and perceived barriers to change	General sustainability
Hathi *et al*. (2023)^69^	Survey (qualitative and quantitative)	Canada	88 ENT surgeons	Assess the attitudes and perceptions of otolaryngologists on environmental sustainability	General sustainability
Lam *et al*. (2023)^70^	Survey (quantitative)	UK	110 members of the public (age range 18-80+) and 100 clinical staff: including two foundation year, six core training, 19 specialty registrar, five associate specialist, 22 consultants, six nontraining post, 10 other (age range 18–69). Variety of specialties, for example general surgery, orthopaedics, anaesthetics, theatre nurses	Determine the attitudes and beliefs of perioperative staff and the public to sustainability initiatives in surgery, and whether differences exist between the two groups	General sustainability
Leppanen *et al*. (2022)^71^	Interviews (qualitative)	Finland	26 nurses: 20 perioperative nurses, six nurse managers. Mean age 47 (range 25–63)	Describe how nurses and nurse managers consider sustainable development principles in their daily work, how well they recognise these principles and how these principles are considered in decision-making in perioperative work	General sustainability
Lim *et al*. (2023)^72^	Survey (quantitative)	Malaysia	163 theatre staff (including surgeons, nurses, and medical students)	Investigate the knowledge, attitude, and practices of OT staff towards sustainable practices	General sustainability
McGain *et al*. (2012)^73^	Survey (qualitative and quantitative)	Australia, New Zealand, and England	780 anaesthetists	Survey views of recycling held by anesthesiologists in either regional or metropolitan and public or private practice	Recycling
Meyer *et al*. (2022)^74^	Survey (quantitative)	US	219 surgeons	Assess perspectives on intraoperative waste and willingness to work to actively reduce this waste	Waste reduction
Petre *et al*. (2020)^75^	Survey (quantitative)	Canada	33 anaesthetists: 26 anaesthesia department chiefs, seven residency programme directors	Survey anaesthesia department chiefs on the current state of environmentally sustainable anaesthesia practice and to understand educational endeavours from anaesthesia residency programme directors	General sustainability
Sürme *et al*. (2022)^76^	Interviews (qualitative)	Turkey	15 nurses. Mean age 38.06 (SD 9.39)	Examine the opinions of nurses working in surgical wards on recycling and medical waste management	Recycling Waste management
Thiel *et al*. (2017)^77^	Survey (quantitative)	US	166 surgeons in obstetrics and gynaecology. Median age 36	Assess the attitudes of physicians and their willingness to participate in efforts to reduce the environmental impact of health services	General sustainability
Tordjman *et al*. (2023)^78^	Survey (quantitative)	France	1092 Anaesthetist, anaesthesia resident and anaesthesia nurse	Evaluate environmentally sustainable anaesthesiology practices in France in 2020 and understand the barriers to their adoption	General sustainability
Yap *et al*. (2023)^79^	Survey (quantitative)	US	205 surgeons, anaesthetists and theatre staff including theatre nurses and scrub technologists	Determine the perceptions of the perioperative staff including attending surgeons, trainee surgeons, and OR staff on the adoption of reusable surgical gowns	Reuasables vs single-use items Waste reduction
Zaw *et al*. (2023)^80^	Interviews (qualitative)	Singapore	23 anaesthesiologists: six medical officers (junior doctors), three residents, three senior residents, two associate consultants, four consultants and five senior consultants. Nine had less than 5 years of anaesthetic experience, seven had 5–10 years, three had 11–15, and four had over 20 years of experience	Address the gap between being interested in implementing environmentally sustainable anaesthetic practices and actually doing so, by analysing barriers and facilitators to green practices among anesthesiologists through the lenses of the Behavioural Change Wheel (BCW) framework	Anaesthesia

### Data synthesis

The results data from each paper were also coded by two of the three researchers (A.A., C.B., and M.B.) to the domains of the TDF. These were then discussed and refined in a meeting of the authors until a consensus was reached. The data were further divided into specific themes within each domain in order to better categorise the data. Each theme was also coded as a barrier or facilitator to behaviour change, or both. The results were tabulated and simple summary statistics used to present the data.

### Quality assessment

Quality assessment was completed using the Mixed Methods Appraisal Tool (MMAT)^81^, due to its relevancy for both quantitative and qualitative studies. Two reviewers (A.A. and C.B.) independently assessed each study using the tool in Excel and discussed any discrepancies until a consensus was reached.

## Results

**Figure 1:**
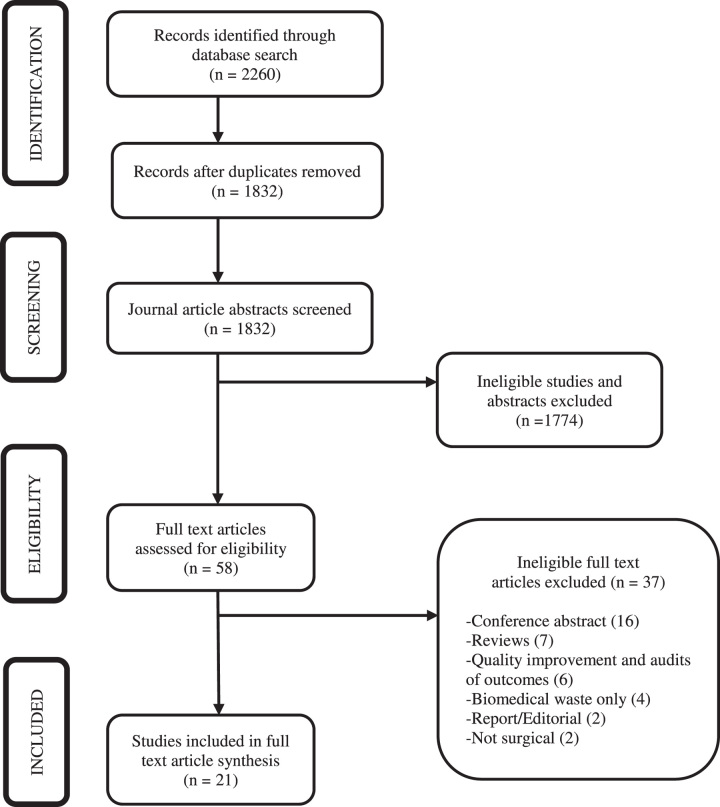
PRISMA diagram.

The search resulted in 2260 studies. Four hundred twenty-eight were duplicates, leaving 1832 articles. One thousand eight hundred thirty-two studies were subjected to title and abstract screening against our inclusion and exclusion criteria. Of these, 58 met our criteria and were subjected to full-text screening. Of these, 37 articles were excluded for the following reasons: 16 were conference abstracts, 7 were reviews, 6 were audits of outcomes and quality improvement projects, 4 articles discussed biomedical waste only, 2 were editorials and 2 were nonsurgical, leaving 21 eligible for inclusion in the final review. Figure 1 shows the number of studies identified through the database search and screening process, and the reasons for exclusion.

### Characteristics of the included studies

Table 3 shows the characteristics of the included studies. The highest number of studies (10) were from the United States. Of the remainder, there were two from Canada and one from each of Australia, the UK and Ireland, the UK only, South Africa, Turkey, France, Malaysia, Singapore, and Finland. Four of the included studies were interviews and the rest (17) were surveys, of which 12 collected quantitative data only. The total number of participants across the studies was 8286, ranging from 15 to 2036 included in each study; however, one study did not report the number of participants.

The studies sampled different staff members, including nurses, surgeons, supporting theatre staff, representatives from an anaesthetic gas machine company, and department chiefs. One study also included members of the public. Often, studies did not differentiate between roles when reporting determinants of behaviour. Many studies did not report the specific role or specialty of the participants; of those that did, eight involved anaesthetists and associated healthcare workers^60,62,66,67,73,75,78,80^, and one study for each of the specialties of cataract related healthcare^63^, obstetrics and gynaecology^77^, ear nose and throat^69^, and ophthalmology^64^. A further study reported the department of each participant, with 11 specialties included, such as vascular surgery, trauma and orthopaedics, and plastic surgery^70^.

Twelve studies aimed to investigate general knowledge, attitudes, and behaviours around sustainability in the workplace^60,64,66–72,75,77,78^. Eight were focused on specific areas of sustainability, either recycling or waste reduction/management or both^61–63,65,73,74,76,79^, and the remaining study investigated anaesthesia^80^. Most studies only reported barriers to change rather than facilitators.

### Quality assessment

Table 4 shows results from the quality assessment of the studies, separated into quantitative descriptive studies and qualitative studies. All studies passed the screening questions and therefore the MMAT tool was suitable to use. All qualitative studies were of strong quality, with the exception of Burbridge *et al*.^62^, where reporting of the results was considered suboptimal and was therefore deemed to be of moderate-weak quality.

**Table 4:** Risk of bias analysis using MMAT.

	Screening questions		Quantitative descriptive studies				
Survey studies	S1. Are there clear research questions?	S2. Do the collected data allow to address the research questions?	4.1. Is the sampling strategy relevant to address the research question?	4.2. Is the sample representative of the target population?	4.3. Are the measurements appropriate?	4.4. Is the risk of nonresponse bias low?	4.5. Is the statistical analysis appropriate to answer the research question?
Ard *et al*. (2016)^60^	Yes	Yes	Yes	No	Yes	No	Yes
Azouz *et al*. (2019)^61^	Yes	Yes	Yes	No	Yes	No	Yes
Chang and Thiel (2020)^63^	Yes	Yes	Yes	Cannot tell	Yes	Cannot tell	Yes
Chang *et al*. (2023)^64^	Yes	Yes	Yes	Yes	Yes	Yes	Yes
Conrardy *et al*. (2010)^65^	Yes	Yes	Yes	No	Yes	Cannot tell	Yes
Fraifeld *et al*. (2021)^66^	Yes	Yes	Yes	No	Yes	No	Yes
Frewen *et al*. (2022)^67^	Yes	Yes	Yes	No	Yes	Cannot tell	Yes
Harris *et al*. (2022)^68^	Yes	Yes	Yes	No	Yes	No	Yes
Hathi *et al*. (2023)^69^	Yes	Yes	Yes	No	Yes	No	Yes
Lam *et al*. (2023)^70^	Yes	Yes	Yes	Cannot tell	Yes	No	Yes
Lim *et al*. (2023)^72^	Yes	Yes	Yes	Yes	Yes	Yes	Yes
McGain *et al*. (2012)^73^	Yes	Yes	Yes	No	Yes	No	Yes
Meyer *et al*. (2022)^74^	Yes	Yes	No	No	Yes	No	Yes
Petre *et al*. (2020)^75^	Yes	Yes	Yes	Cannot tell	Yes	No	Yes
Thiel *et al*. (2017)^77^	Yes	Yes	No	No	Yes	Yes	Yes
Tordjman *et al*. (2023)^78^	Yes	Yes	Yes	Yes	Yes	No	Yes
Yap *et al*. (2023)^79^	Yes	Yes	Yes	Yes	Yes	No	Yes
	Screening questions		Qualitative studies				
Qualitative studies	S1. Are there clear research questions?	S2. Do the collected data allow to address the research questions?	1.1. Is the qualitative approach appropriate to answer the research question?	1.2. Are the qualitative data collection methods adequate to address the research question?	1.3. Are the findings adequately derived from the data?	1.4. Is the interpretation of results sufficiently substantiated by data?	1.5. Is there coherence between qualitative data sources, collection, analysis and interpretation?
Burbridge *et al*. (2019)^62^	Yes	Yes	Yes	Yes	Cannot tell	No	Cannot tell
Leppanen *et al*. (2022)^71^	Yes	Yes	Yes	Yes	Yes	Yes	Yes
Sürme *et al*. (2022)^76^	Yes	Yes	Yes	Yes	Yes	Yes	Yes
Zaw *et al*. (2023)^80^	Yes	Yes	Yes	Yes	Yes	Yes	Yes

Almost all the quantitative studies were deemed to be of strong or moderate-strong quality; all studies had a clear research question(s) and the data collected allowed the research question(s) to be answered. Additionally, the sampling methods were deemed relevant to the research question(s), and measurements and statistical analysis were appropriate. However, most were deemed either to have samples which were not representative of the target population, or this was unclear. Similarly, the nonresponse bias was not appropriately addressed in nearly all the studies. The only exception was Meyer *et al*.^74^, which was deemed to be of moderate-weak quality.

### Determinants according to the TDF

The TDF domains are described below, and summarised in Tables 5 and 6. Of the 10 domains observed, the most commonly reported were ‘knowledge’ (18 papers) and ‘environmental context and resources’ (16 papers). We coded 18 different themes within the ten domains of the TDF. Eleven of these were barriers, five were both a barrier and a facilitator, and two were facilitators. The domains are discussed in turn below. The TDF domains not observed in these studies were: skills, optimism, behavioural regulation, and goals.

**Table 5:** Summary of the included studies and TDF domains covered in this review.

	TDF domain									
Study	Knowledge	Environmental context and resources	Intentions	Beliefs about consequences	Social influences	Social/ professional role and identity	Emotion	Memory, attention and decision processes	Reinforcement	Beliefs about capabilities
Ard *et al*. (2016)^60^	X	X	X		X					
Azouz *et al*. (2019)^61^	X	X								
Burbridge *et al*. (2019)^62^	X	X		X						
Chang & Thiel (2020)^63^	X	X	X	X			X	X		
Chang *et al.*(2023)^64^		X	X	X						
Conrardy *et al*. (2010)^65^	X									
Fraifeld *et al*. (2021)^66^	X									
Frewen *et al*. (2022)^67^	X					X				
Harris *et al*. (2022)^68^	X	X	X	X	X		X			
Hathi *et al*. (2023)^69^	X	X	X		X				X	
Lam *et al*. (2023)^70^		X	X	X	X	X				
Leppanen *et al*. (2022)^71^	X	X			X	X				
Lim *et al*. (2023)^72^	X		X							
McGain *et al*. (2012)^73^	X	X	X	X	X					
Meyer *et al*. (2022)^74^	X	X	X				X			
Petre *et al*. (2020)^75^	X	X	X		X					
Sürme *et al*. (2022)^76^	X	X								
Thiel *et al*. (2017)^77^				X				X		
Tordjman *et al*. (2023)^78^	X	X	X		X					
Yap *et al*. (2023)^79^	X			X						
Zaw *et al*. (2023)^80^	X	X		X	X	X				X

X indicates the TDF domain is covered by the relevant study

**Table 6:** TDF domains, themes, and examples.

TDF domain (*n*=number of studies reporting this domain)	Coded themes (*n*=number of studies reporting this theme)	Examples	Barrier or facilitator	Who gave this barrier
Knowledge (*n*=18)	Knowledge of sustainability practices (*n*=14)	When queried about the justification for separating regulated medical waste, staff members were not able to verbalise what is considered to be regulated medical waste and what is not	Barrier	Surgeons and surgical technologists
	Knowledge of sustainability context (*n*=8)	‘I don’t have much awareness on how bad is the harmful effect of that. And hence, I feel that the little things I do may not have much impact, hence, I continue my practice as what it is’.	Barrier and facilitator	Anaesthesiologists
		None of the participants had heard about the Sustainable Development Agenda 2030 or about the UN’s Sustainable Development Goals		Nurses and nurse managers
		‘From the research that I did, it seems like anaesthesia itself does seem to have a fair share of in terms of pollution, from anaesthetic waste gases, from the single-use plastics that we use, even to the drugs in terms of the disposal of drugs itself, causing impact to the environment’.		Anaesthesiologists
Environmental context and resources (*n*=16)	Personnel shortage and workload (*n*=11)	Half of the nurses stated that medical wastes were thrown indiscriminately due to the high workload and lack of staff	Barrier	Nurses
		‘People are more preoccupied [with] starting a list on time, finishing the list, making sure patients are… I mean, some patients are really sick. So they were more interested in keeping patients hemodynamically stable than trying to think of [the] environmental impact of the anaesthetic … so environmental concerns are perhaps not at the top of their minds doing the list’.		Anaesthesiologists
	Inadequate facilities or environment for appropriate waste disposal (*n*=8)	When asked what the greatest barrier to recycling was, the most common one was inadequate recycling facilities (49%)	Barrier	Anaesthetists
		Inconvenient bin location		
	Availability of financial resources (*n*=6)	Around half of respondents reported perceived additional barriers of cost	Barrier	Surgeons
	Equipment design (*n*=3)	The canister was designed to remain intact and not to be recycled despite having the recycling symbol	Barrier	Anaesthetic absorber product representatives and manufacturers, anaesthetists, anaesthesia technicians, nurses, waste management personnel
	Process or organisational constraints (*n*=2)	Cited as having the highest impact by the surgeon respondents were regulatory agencies (82%) or facility regulations (74%) limiting surgeon discretion for reusing supplies	Barrier	Surgeons
Intentions (*n*=11)	Problem awareness and willingness to change (*n*=11)	The majority were willing to make changes to their personal clinical practice, and around half to become ‘green champions’ or to join a focus group	Facilitator	Surgeons
		The majority of department chiefs indicated there was either interest or active efforts to expand sustainability education programs		Heads of department and programme directors
Beliefs about consequences (*n*=9)	Concerns regarding safety (*n*=9)	‘[Our] practices have somewhat been environmentally unfriendly like the single-use syringes… but it is an attempt to maintain sterility and patient safety… I think, for me, it’s more important that patient safety is upheld. I don’t really think of environmental consequences’.	Barrier	Anaesthesiologists
	Belief of lack of impact of change (*n*=1)	‘[I]f I make all the effort, I would like to know that it is actually going to where it should go, which is to the recycling plant, rather than going to the rubbish dump or the incinerator, along with all the rubbish. If it’s going to go that way, why do I want to make all the effort to separate it?’	Barrier and facilitator	Anaesthesiologists
Social influences (*n*=9)	Lack of leadership / organisational mandate or support (*n*=9)	Less than half of surgeons felt that they had the support of their department (51/130; 39%), colleagues (65/130; 50%) or NHS trust (46/130; 44%)	Barrier	Surgeon and nurses
		Perioperative nurses considered that they have limited opportunities to influence to the usage of disposable products in surgeries. ‘In my position as a nurse… If the surgeon wants something, I can’t do anything about it’.		
	Influence of more senior clinicians (*n*=1)	“When I’m with my seniors, whatever they do, I will just follow suit… definitely influenced a lot by my seniors’ practice’.	Barrier and facilitator	Anaesthesiologists
Social/professional role and identity (*n*=4)	Professional responsibility for sustainable practices (*n*=4)	Participants felt garbage sorting was not part of their job description	Barrier and facilitator	Nurses and nurse managers
		92% of South African anaesthetic providers agreed or strongly agreed that the environmental impact of anaesthesia-related products, agents and equipment should be taken into account when making clinical decisions		
Emotion (*n*=3)	Concern about climate change (*n*=2)	A total of 1139 respondents (91%) expressed concern about global warming and climate change	Facilitator	Surgeons
	Lack of concern about sustainability (*n*=1)	When asked about the barriers to reducing operating theatre waste, respondents identified lack of awareness of OR waste (67%) most frequently, followed by lack of concern about OR waste (64%)	Barrier	Surgeons
Memory attention and decision processes (*n*=2)	Surgeon decision and preferences (*n*=2)	Only 20% preferred the reusable instruments over disposables	Barrier	Surgeons and nurses
Reinforcement (*n*=1)	Incentives (*n*=1)	The most prevalent barriers to environmental sustainability in the OR were lack of incentives	Barrier	Surgeons
Beliefs about capabilities (*n*=1)	Capable to decide on environmentally sustainable option (*n*=1)	‘[L]ike switching from Des to Sevo, there is no new capital expenditure to buy new drugs or anything like that. It’s already right in front of you, you just turn it on or turn it off… so it’s just whether you want to change your own practice’.	Barrier and facilitator	Anaesthesiologists

### Knowledge

Knowledge was the domain reported most frequently, with 18 (86%) of the studies citing barriers relating to a lack of knowledge. These were further divided into the themes of ‘knowledge of the sustainability context’, and ‘knowledge of sustainable practices’ in the workplace. ‘Knowledge of the sustainability context’ as a barrier included not knowing that there was an environmental impact of inhaled anaesthetics^60^ or that there were sustainability-related goals^71^. It was also a facilitator, for example in Zaw *et al*.^80^, some anaesthetists were aware of the environmental impact of their work^80^. ‘Knowledge of sustainable practices’ was the most cited theme overall, with fourteen (67%) of the studies identifying a lack of this knowledge as a barrier. Some examples of this theme included not knowing whether waste is recyclable^61^ or which bin was appropriate for which item of waste^60,62,65^.

### Environmental context and resources

Issues of environmental context and resources were cited by 16 (76%) of the studies. Within this domain five themes were identified. The most frequent was ‘personnel shortage and workload’ (11 studies; 52%). This was often related to workload and time pressures in the operating theatre, which meant that, for example, waste was ‘thrown indiscriminately’^76^.

A second environmental context issue was ‘inadequate recycling facilities’. This barrier was cited in eight studies (38%) and included ‘inconvenient bin location’^61^ or a lack of recycling facilities^73^. ‘Availability of financial resources’ was a further barrier within this domain (6 studies; 29%). In these studies, participants noted that their hospitals did not have the funding or budget to research or implement programmes to improve sustainability^62,68,70,73,75,78^. ‘Equipment design’ was referenced as a barrier in three studies (14%). These studies reported the design of packaging being wasteful^63^, that an anaesthetic gas canister was difficult to take apart in order to recycle each piece^62^, and that single-use items were packaged in a way that created unnecessary waste^64^.

There were also two studies (10%) which noted ‘process or organisational constraints’ involved in reducing environmental impact—Chang and Thiel^63^ found that most surgeons in their survey reported not having enough influence over reusing supplies. Rather, regulatory agencies and facility regulations have more authority regarding whether materials can be reused or need to be thrown away; this was reported in a further study in 2023^64^.

### Intentions

Eleven studies (52%) reported intentions as a facilitator to sustainable behaviour. For example, in Chang and Thiel^63^, 93% of participants felt that they should develop approaches to reduce waste and in McGain *et al*.^73^, 90% said they wished to recycle at work. Some studies indicated that this intention had gone further, for example in Harris *et al.* ^68^, surgeons felt that they were making sustainable behaviour changes individually rather than being aided by the wider hospital, through switching to reusable items or repairing broken equipment rather than replacing it.

### Beliefs about consequences

There were nine studies (43%) which reported this barrier. Many were related to ‘concerns about safety’, which was generally patient safety. For example, 95% of physicians in Thiel *et al*.^77^ supported waste reduction in OTs, with the caveat that this did not impact patient care. The participants in some studies also felt specifically that single-use products were safer^63,77^. Zaw *et al.*’s^80^ qualitative study illustrated that staff’s priority is patient care and maintaining high levels of sterility, which can result in more environmentally impactful practices—‘a necessary evil’ to providing good-quality care. For example: ‘it is an attempt to maintain sterility and patient safety … I think, for me, it is more important that patient safety is upheld. I do not really think of environmental consequences’^80^.

This theme did not only relate to patient safety but also staff safety; in one study which looked at anaesthetic absorbers, participants noted that if they take the absorbers apart (required for recycling), they risk exposure to dangerous soda-lime dust^62^.

A final theme relating to beliefs about consequences was participants’ beliefs about how impactful any changes they made would be. Participants in one study noted that if it was not specified that waste would actually be recycled after being taken away, they did not believe it was necessary to separate it thoughtfully. Similarly, others in the same study felt that any effort made on their part would be ‘like a drop in the ocean’; there was not enough benefit to making environmentally friendly changes^80^.

### Social influences

Nine studies reported social influences as a barrier in the theme of ‘lack of leadership/organisational mandate or support’ (nine studies; 43%). In Petre *et al*.^75^, participants felt that there was a lack of institutional or departmental support for sustainability measures and either no clear mandate from leadership in the hospital or no mandate at all, and these findings were repeated in other studies^60,68–70,73,78^. Leppänen *et al*.^71^ also found that nurses were not supported by surgeons when suggesting less wasteful behaviours.

One study (5%) noted social influences as either a barrier or facilitator to behaviour change in the operating theatre. In this study, junior anaesthesiologists said that they would follow what their seniors were doing, whether or not it would have a negative impact on the environment^80^.

### Social/professional role and identity

Overall, ‘social/professional role and identity’ was a behavioural determinant in four studies, all coded under ‘professional responsibility for sustainable practices’ (19%). This theme acted as both a facilitator and a barrier. In Leppänen *et al.* ^71^, sorting waste ‘was not felt to be part of (nurses’) job description’, however, Frewen *et al.* ^67^ reported that 92% of anaesthetic providers surveyed agreed that they should consider the environmental impact of the products they use in their work when making clinical decisions.

### Emotion

Emotion-related determinants were reported in three studies (14%). These came within a theme of ‘concern about climate change’, which was a facilitator to behaviour change: for example, in Harris *et al.* ^68^, 94% of participants reported concern about global warming to varying degrees. Conversely, one study (Meyer *et al.* ^74^) reported a lack of concern about sustainability as a barrier. This was more specifically a lack of concern about operating theatre waste, rather than climate change in general^74^.

### Memory, attention, and decision processes

Two studies (10%) mentioned barriers relating to ‘surgeon decisions and preferences’. Thiel *et al.* ^77^ found that only 20% of surgeons preferred reusables in their sample of 166 participants. In Chang and Thiel’s^63^ survey of 1634 participants, surgeon preference was not a strong driver of using disposable products.

### Reinforcement

One study (5%) mentioned barriers relating to incentives. This survey found that the most common barriers to environmental sustainability in OTs were ‘lack of incentives’^69^.

### Beliefs about capabilities

Beliefs about capabilities was discussed in one study (5%). In Zaw *et al*.^80^, clinician preference could be either a barrier or facilitator to reducing the environmental impact. Whether or not to use a less impactful anaesthetic gas was solely down to anaesthesiologist preference, and therefore they would be able to use a more environmentally friendly gas if they chose to^80^.

## Discussion

This review collated the barriers and facilitators for reducing the environmental impact of OTs. Our findings suggest that many healthcare professionals would like to, or intend to make more sustainable choices and adopt ‘green’ behaviours in their work, but these intentions are impeded by various barriers. This review found that the main barriers to sustainable practices in OTs were knowledge and environmental context and resources.

Knowledge was the most common obstacle cited for adopting green behaviours by healthcare professionals in general. In particular, there was an evident lack of knowledge of the sustainable practices in the workplace, including knowledge of which items to recycle, effective waste-segregation, and appropriate disposal of waste. Studies in the literature have proposed and trialled interventions which addressed this barrier. For instance, a study by Southorn *et al.* ^82^ showed that staff education coupled with bin labelling can help reduce carbon footprint by up to 75%. Similarly, a study by Wyssusek *et al.* ^83^ resulted in a significant reduction in the amount of regulated medical waste and a significant increase in the amount of recyclable waste following an educational programme for waste-segregation, highlighting the importance of continued sustainability education and training. The importance of sustainability training and education has been acknowledged by the Medical Schools Council in the UK, which outlined the need for an ‘Education for Sustainable Healthcare’ curriculum^84^. Due to the rapid advancements in healthcare practices, and the change in guidelines, regulations and recommendations, continued education and retraining for qualified healthcare professionals is fundamental.

Furthermore, our review also demonstrated that healthcare professionals reported a significant lack of ‘knowledge of environmental context’, including the impact of practices on the environment. This barrier affects staff’s ability to make an informed and environmentally friendly choice regarding surgical instruments or practices, when the chance arises. Moreover, lack of knowledge could be a driver of the ‘just in case’ culture, where surgeons may ask theatre staff to open (and later discard) single-use equipment ‘just in case’ they need it—which is highlighted in The Royal College of Surgeons of England (RCSEng) planetary bulletin^85^.

As with ‘knowledge of sustainable practices’, staff education could help increase healthcare professionals’ knowledge of the environmental impact of their work. Since 2000, the Centre for Greening the NHS at the Institute of Health Sciences, Oxford, UK has been advocating for the use of Life Cycle Assessment methods to quantify the carbon footprint of surgical equipment, and to guide procurement on strategic and operational levels^86^. Another way to tackle the lack of knowledge of environmental impact in healthcare behaviours could be the eco-labelling of products and supplies to steer surgery towards sustainability. According to the Global Eco-labelling Network an “ecolabel is a label which identifies the overall environmental preference of a product or service within a specific product/service category based on life cycle considerations.” Eco-labelling could also influence surgeons’ choices and help drive greener procurement—one of the top three carbon hotspots in healthcare- through a bottom-up approach^87^. Surgeons, who are frequently cited as decision-makers in OTs, should be made aware of the carbon footprints of the instruments they can choose from to complete a task, and the environmental cost of opened unused equipment. Studies show that eco-labelling significantly affects consumers’ green purchasing intentions and attitudes^88^, and appears to be effective in reducing the carbon footprint of several industries^89^. Although it has not been widely adopted in healthcare, there have been some interventions which have found that labelling equipment in the healthcare setting with their carbon emissions (e.g. different anaesthetic gases) changes physicians’ behaviour, thus reducing environmental impact^4,90,91^. Eco-labelling theatre equipment may empower the staff to make informed choices, reduce the ‘just in case’ culture in OTs (move to a ʻjust in timeʼ culture), and influence procurement through a bottom-up approach.

The second most commonly cited barrier in this review was from the domain of environmental context and resources, most notably personnel shortage and workload, and inadequate facilities. Nurses and other healthcare staff often described a shift in priorities whereby sustainable behaviours are side-lined due to workload—presumably as patient safety and service provision take priority. The RCSEng planetary bulletin^85^ stresses the paradoxical threat posed by healthcare greenhouse gas emissions, specifically the relationship between healthcare and emerging zoonotic diseases linked to climate change. Given that there may be an immediate threat to life, it is only natural to prioritise service delivery over long-term environmental consequences. Indeed, prioritising sustainable choices in a stretched healthcare system can be challenging; however, we recommend that organisations recognise this barrier and work within their limits to break this cycle, in order to improve public health in general^92^. The application of both behavioural science and design is recommended to support the design of effective and practical solutions to facilitate sustainable behaviours in OTs.

In the past, medicine, and surgery in particular, were often considered hierarchical workplaces^93^. Despite a cultural shift towards a less hierarchical workplace, reports still show an imbalance of power in healthcare^94,95^. Our results show that hierarchy, as well as a lack of leadership or organisational support, are significant barriers to the adoption of sustainable practices. Nurses felt their suggestions about less wasteful practices did not matter, and that often surgeons ‘rule’. On the other hand, surgeons felt unsupported by their departments, managers and lacked organisational mandates to adopt greener practices. It is important for both organisations and individuals to recognise these barriers and adopt practices that promote social and organisational values related to sustainable OTs. This could be achieved by developing and adopting a flexible framework relating to Communities of Practice (CoPs) in green theatres^96^. The concept of CoPs, which was first proposed in 1991 by Lave and Wenger, brings together individuals with a shared interest and has been shown to improve organisational performance in the business industry and has gained recognition for adoption in healthcare. It has been redefined in 2002 by Wenger *et al.* ^97^ as a tool to share knowledge and to innovate, characterised by a shared domain, a group of people in the organisation, and a shared repertoire. CoPs vary in form and purpose, however, a systematic review by Ranmuthugala *et al.* ^96^ demonstrated growing evidence supporting the use of CoPs as a tool to improve practice and facilitate evidence-based changes in medicine.

The TDF domain ‘beliefs about consequences’ was also coded in this review. In five studies, ‘concerns about safety’ was reported as a barrier to the adoption of sustainable practices in OTs. Participants were concerned that practices such as switching to reusable instruments or textiles as opposed to consumables, which are the second largest source of greenhouse gases in OTs^10^, could compromise their safety, patients’ safety or the safety of other colleagues^63,77^. However, the literature does not support the hypothesis that disposable items are safer^98^. In fact, Thiel *et al*.^99^ demonstrated that phacoemulsification procedures in India produce 6% of the CO_2_-equivalent units produced by the same procedure in the UK by using reusable instruments and materials, while maintaining similar infection rates. Identified studies showed inconsistent results on staff preference. Thiel *et al.* ^77^ showed that only 20% of surgeons preferred reusable items, and previous research observed that one of the main reasons for preferring single-use equipment by surgery staff is convenience. However, it is not clear how much surgeons’ Preferences influence practice. A survey of 1600 participants by Chang and Thiel^63^ showed that surgeons’ preference was not a strong influence. Instead, 70–80% of participants cited hospital regulations and supply manufacturers as the driving force of equipment choice^63^. More research is needed to establish the drivers behind the reluctance to use reusable alternatives in surgery. More importantly, interventions aimed at encouraging the switch to reusable equipment should target all stakeholders, including the managers, and should factor the ‘Triple Bottom Line’ of environmental, social (highlighting that safety is not impacted), and financial impacts to promote sustainable transformation across the board^100^.

Our study had some key limitations. To start with, only English language studies were searched for, which may limit the behavioural determinants found. Therefore, we cannot say that these determinants represent all countries and OTs. Many countries were not represented in these studies, including more remote areas in developing countries, which may face very different barriers to sustainability. However, a strength of the review is that all parts of the review process were completed by multiple researchers. For example, the final coding of TDF domains and themes was iterated in discussion between six researchers, resulting in robust findings. Furthermore, using the TDF as a method of organising the barriers and facilitators will facilitate more systematic and rigorous design of interventions to address the identified determinants, using frameworks for mapping behavioural determinants to intervention functions (broad types of interventions) and behaviour change techniques^48,51,101^.

In terms of the limitations of the included studies, almost all quantitative studies had quality issues with having a nonrepresentative sample and the risk of nonresponse bias. There is therefore a high chance that the determinants in this review do not reflect the views of even the intended target populations. Despite including several different actors within the surgical pathway (i.e. nurses, doctors, nurse managers, waste management personnel), survey studies in particular did not differentiate between the views of different healthcare professionals when reporting results, with findings only reported in aggregate. Therefore, it was not possible to identify differing determinants in different groups, which is important given the different environmental behaviours each may have an influence on, and in terms of informing the design of interventions appropriate to the decision-makers for each environmental behaviour.

Furthermore, none of the included studies used a framework or theory to inform the investigation of barriers and facilitators. Therefore, it is not clear whether the TDF domains not identified in the review (e.g. skills and behavioural regulation) were not relevant due to not being determinants of environmental behaviours, or simply not identified because they were not investigated. Additional detail in reporting findings on determinants of behaviour would help the research to be more useful when designing interventions.

Future research into determinants of environmentally sustainable behaviours in OTs should include qualitative methods, such as interviews and ethnography studies, to gain a more in-depth and nuanced perspective of the barriers and facilitators to different target behaviours. Separating results according to professional role will enable an understanding of which determinants are most important for the professionals who affect each environmental behaviour (e.g. some choices may be made by nurses, and some by surgeons). The use of a behavioural theory or framework in research on determinants will ensure comprehensibility, so no potentially relevant determinants are neglected, and will also support subsequent intervention design. The findings from this review and future research should be used to inform the design of interventions to improve sustainable behaviours within OTs, by overcoming barriers (e.g. knowledge, context and resources, and beliefs about consequences) and harnessing facilitators, (e.g. emotion). Participatory, co-design methods should be used, to ensure that interventions are acceptable and practical to integrate into the operating theatre workflow, and will be effective given contextual barriers that are not feasible to mitigate (e.g. time and personal shortages).

### Conclusions

This review found that the main barriers cited in the literature to green surgery are lack of knowledge (of sustainable practices or sustainability goals) and context and resources (e.g. staffing, time pressures, or inadequate recycling facilities). Lack of organisational support was also cited as a barrier, yet there was evidence of intentions to make proenvironmental changes. Interventions should be co-designed with theatre staff to address these determinants in a feasible and effective way, for example by training theatre staff on sustainable behaviour and creating a ‘green culture’. However, there are limitations to the available evidence, and we recommend additional in-depth work, based on behavioural science frameworks, to give a deep and nuanced understanding of the barriers and facilitators from various stakeholders in the operating theatre context. More detailed and specific knowledge would better inform interventions facilitating sustainable behaviours with minimal effort (e.g. replacing single-use equipment with reusable) or by facilitating informed decisions (e.g. labelling packages with explicit waste instructions, for example: recycle). Solutions informed by a full understanding of the most important contextual barriers and facilitators are more likely to be effective, and will also address the inevitable barriers of time, personnel shortages, and the need for convenience.

## Ethical approval

Ethical approval not needed - systematic review.

## Patient consent

Not applicable.

## Sources of funding

This work was supported by the Medical Research Council grants [MR/X011720/1, MR/X502959/1], and a National Institute for Health and Care Research (NIHR) fellowship.

## Author contribution

A.A., M.B., J.W.B., D.L., T.P., P.D., and G.J.: contributed to conception and design of the study; A.A., C.B., and M.B.: contributed to acquisition and analysis of data; A.A., C.B., M.B., G.J., P.D., and T.P.: contributed to interpretation of data; C.B. and A.A.: wrote the first draft of the manuscript; T.P., P.D., and G.J.: wrote sections of the manuscript. All authors contributed to manuscript revision, read, and approved the submitted version.

## Conflicts of interest disclosure

The authors declare that the research was conducted in the absence of any commercial or financial relationships that could be construed as a potential conflict of interest.

## Research registration unique identifying number (UIN)

reviewregistry1704.

## Guarantor

All contributing authors.

## Data availability statement

The studies included in this review are publicly available. Template data collection forms, data extracted from included studies, and data used for analysis are available upon request.

## Provenance and peer review

Not commissioned, externally peer-review.
